# Metallic Crowns

**Published:** 1883-06

**Authors:** Wm. N. Morrison

**Affiliations:** St. Louis, Mo.


ARTICLE VII.

### METALLIC CROWNS.

BY WM. N. MORRISON, D. D. S., OF ST. LOUIS, MO. (Continued.)

Place the crown on the root, apply the dam, dry, and fill the crown with amalgam through the opening left in the crown on the masticating surface. I usually fill the crown partially before placing it in position to save time, but the end of the root must be dry by using the dam or napkins before the crown is adjusted; if this is done be sure the articulation is correct.

The shell should be closely burnished to the neck of the tooth, and a gold filling may be introduced in the opening at another sitting if desired.

Dr. Gilmer: A method which I have adopted in making gold crowns, which secures a perfect fit and articulation, is as follows: Prepare the root as described by Dr. Brophy, only I think a small fissure bur will aid very materially in the straightening of that part of the root to which the crown is to be attached. Take a small silver or gold wire, put it about the root, bringing the ends together and twisting till it fits the root tightly, remove, place on a tapering mandrel and secure the exact prize, mark the mandrel at the point reached by the wire, cut a very thin strip of very thin platina one-eighth inch or more in width, according to the case, bend around the mandrel at the place marked and cut the exact size, bring the two ends together and solder with pure gold. This band is the exact size of the root. Next, cut a strip of pure gold plate, in width equal to the length of the desired crown; make of this a barrel which will just fit over the outside of the platina band, solder this with a high grade of solder so that in future soldering it will not be undone. Apply the platina band to the root, place the gold barrel on it and press up until the gum is nearly or quite reached; trim the grinding surface to a proper articulation with the antagonizing tooth, shape the barrel to correspond with the neighboring teeth, strike up a suitable cap, remove the barrel and solder the cap to it, replace on the root, cause the patient to close the teeth when the crown will slip up on the platina band as much further than before as the thickness of the cap; mark the platina at the point reached by the crown, then remove both together if possible, if separated, replace the crown on the platina band up to the mark, solder with a lower grade of solder than either of the previous times, polish, drill hole in the grinding surface for escape of excess of cement and apply, directing the patient to close the teeth until a natural articulation is obtained. When the cement is hard fill the drill hole, and the work is completed.

Platina is employed for the band instead of gold because it may be used much thinner without danger of melting down in soldering. Such a crown can be made in a very short time and obviates the necessity of impressions and models.

Dr. Talbot: I wish to direct the attention of the society to the fact that the author of this paper just read, is the original inventor of these gold crowns. I once made some effort to trace out the origin of this method of operating and found out the wrong man, namely, Dr. Beers, of California, whose patent I thought at that time to have been the source of Dr. Richmond's knowledge of the operation.

I found that Dr. Beers, of California, had practiced the method and obtained a patent upon it sometime prior to Dr. Richmond but it is evident now that Dr. Beers was sometime later than Dr. Morrison, and it appears probable that he derived his knowledge of it from the article in the *Missouri Dental Journal* which has been quoted by Dr. Morrison in his paper. I wish to make this explanation and acknowledgement here because of my previous effort to have the origin of this important operation credited to the right man, having gone so wide of the mark.

The preparation of the root and the method of attaching the crown are the important points in the operation. It is necessary that conical roots should be cut so that their sides are straight and parallel up to the process. (I use hoe-shaped instruments to effect this.) If it is not done and the exposed end of the root is the largest part of it, the upper part of the band will not fit close. For the support of the crown the pulp-chamber and root-canal should be enlarged somewhat and well under-cut, and it is desirable to use a cement that will *stick* to both crown and root. The various forms of oxy-phosphate of zinc are very suitable for this purpose. A loop or hook may be soldered to the inside of the crown that will reach down into the pulp-chamber or root-canal. I sometimes use a common steel screw such as carpenters use for wood-work. The sharp, coarse thread cuts its way in the dentine and holds very firmly in the root, and the large flaring head extending inside the crown holds well in the cement.

Dr. Morrison: It occasionally happens that we find a lower molar with the crown broken off and both the roots firm and in good condition. In a case like that I would fit a collar or band to each of the roots separately, and after removing them solder them together in proper relative position, then replace and proceed to make a crown as in an ordinary case. I do not put the band or ferrule upon front roots when adjusting a plate front tooth. It is almost certain to be unsightly and is seldom necessary.—*Trans. Illinois State Dental Society, 1882.*

